# Proposal for Lines of Research Into Consumer Behavior: Examples in the Tourism Industry

**DOI:** 10.3389/fpsyg.2020.00064

**Published:** 2020-02-19

**Authors:** Juan Jose Blazquez-Resino, Santiago Gutiérrez-Broncano, Mario Arias-Oliva

**Affiliations:** ^1^Department of Business Administration, Faculty of Social Science, University of Castilla La Mancha, Ciudad Real, Spain; ^2^Department of Business Management, Faculty of Business and Economics, Rovira i Virgili University, Reus, Spain

**Keywords:** active attitudinal loyalty, passive attitudinal loyalty, behavioral loyalty, tourist destination image, tourist loyalty

## Introduction

Departing from the high relevance that loyalty plays in the promotion of tourist destinations, the study of the variables that result in loyalty is key. For this reason, there are myriads of studies devoted to the analysis of the variables that result in loyalty, especially considering either or both attitudinal and behavioral loyalty; in turn, relatively less frequent are the studies that also/instead consider active and passive loyalty. Within this research line, we go one step further and propose the importance of focusing on the study of the variables that are conducive to attitudinal and behavioral loyalty; moreover, within attitudinal loyalty, we also acknowledge the distinction of two further types of loyalty, that is, active attitudinal and passive attitudinal loyalty. This opinion paper aims at adding knowledge to the field of consumer behavior in tourism, proposing the importance of studying the variables that are able to drive loyalty in a very differentiated way (active attitudinal loyalty, passive attitudinal loyalty, and behavioral loyalty).

## Proposing a New Research Line

Loyalty is a key variable in all economic sectors and industries, so there is no doubt that looking for loyalty is a key priority. In fact, it has been largely proven how loyalty is essential for achieving key company indicators such as long-term competition, profitability, and survival (Jacoby and Chestnut, 1978; Dick and Basu, 1994; Garbarino and Johnson, 1999; Uncles et al., 2003; Rundle-Thiele, 2005; Kim and Li, 2009). In the tourism industry, loyalty is generally regarded as the best predictor for future tourist behavior, as well as a source of success in the market, in addition to providing competitive superiority (Kim and Brown, 2012; Sun et al., 2013; Gursoy et al., 2014; Maghsoodi et al., 2016; Almeida-Santana and Moreno-Gil, 2018; Cossío-Silva et al., 2019). Hence, a key objective for tourist destinations is to attract and retain their target market (Gursoy et al., 2014; Cossío-Silva et al., 2019). These considerations are of special relevance for countries such as Spain, where tourism is one of the main industries, and the economy relies, to a great extent, on tourism (Balaguer and Cantavella-Jorda, 2002; Camisón et al., 2016).

In the marketing literature, several research studies showed how within loyalty, a further distinction between attitudinal loyalty and behavioral loyalty could be made (e.g., Day, 1969; Jacoby, 1971; Jacoby and Kyner, 1973; Lutz and Winn, 1974; Dick and Basu, 1994; Yoon and Kim, 2000; Bowen and Chen, 2001; Chaudhuri and Holbrook, 2001; Lam et al., 2004; Söderlund, 2006). In general, while attitudinal loyalty refers to positive attitudes held by customers toward a particular brand or store, behavioral loyalty refers to repeat purchases by a customer at a specific brand or store (Day, 1969; Dick and Basu, 1994). While it is common to encourage the design of strategies to boost both types of loyalty, it has been observed how sometimes, attitudes might not necessarily lead to repeat patronage. In fact, previous research suggested that attitudinal loyalty not in the presence of re-patronage behavior, and re-patronage not in the presence of attitudinal loyalty, could be conceptualized (Day, 1969; Dick and Basu, 1994; Reynolds and Beatty, 1999). These latter phenomena are sometimes due to custom, chance, or other factors (Day, 1969). In either case, it is very important to refer to both types of loyalty, that is, attitudinal and behavioral loyalty, as two separate constructs, despite the fact of such constructs being inter-related (Dick and Basu, 1994; Bemmaor, 1995; Chandon et al., 2005; Liu, 2007). This perspective is also acknowledged in the tourism literature, where a vast number of studies (e.g., Faullant et al., 2008; Wang et al., 2010; Kursunluoglu, 2011; Forgas-Coll et al., 2012; Prayag and Ryan, 2012; Zhang et al., 2014; Llodrà-Riera et al., 2015) also consider loyalty as a two-dimensional variable, that is, a variable that consists of two separate and inter-related constructs of both attitudinal and behavioral loyalty.

Relatively less analyzed is the further distinction between two other types of loyalty, that is to say, active loyalty and passive loyalty. One of the studies that considered this distinction in the services literature is the research of Ganesh et al. (2000). In this work, loyalty could be considered as either active or passive depending on the predisposition of clients to collaborate with the company. From this point of view, active loyalty was then conceptualized as word-of-mouth communication (WOM), requiring an active compromise reflecting the emotional bonds with the client. Sharing this perspective, Kandampully et al. (2015) suggested that active loyalty was exhibited by those clients that had both a firm compromise and a strong will to serve as ambassadors of the brand, supporting the products and services of the company with a positive WOM. In this regard, social media technologies fostered the development of research oriented to assess both active and passive loyalty. For example, Van Asperen et al. (2018) considered two types of clients' participation in social media: the consumption of social media as passive participation and contribution in social media as active participation. This differentiation could be the key to understand why a recommendation succeeded or, the other way around, failed.

There is no doubt that this proposed research line is of key importance. Departing from the high relevance that loyalty plays in the promotion of tourist destinations, given its connections to long-term profit variables such as long-term competitiveness, profitability, and survival, the study of the variables that result in loyalty is key, especially in countries such as Spain, where tourism is without a doubt the main industry, and the economy relies, to a greater extent, on tourism (Nowak et al., 2007). Spain's economic growth has been positively affected by the persistent expansion of inbound tourism in recent decades (Sokhanvar, 2019). For example, the results obtained in 2018 in terms of the number of foreign visitors to this country were 89,856 million, a 1.1% increase in arrivals over the previous year, which represents an increase in international tourist spending by 3.3% (Ministerio de Energía y Turismo, 2019). These data make Spain as the second country in the world in terms of foreign tourist arrivals (World Tourism Organization, 2019).

## Discussion

Loyalty is often measured by the joint use of its behavioral and attitudinal components. In some markets, such as tourism, repeat visits (behavioral loyalty) may be limited due to other variables, such as “search for variety.” However, a tourist who does not repeat a visit to the same destination may have an important attitudinal loyalty toward that destination and be willing to strongly recommend his visit. Therefore, this opinion paper has been aimed at showing how in the field of consumer behavior in tourism marketing, it is still possible to propose new future lines of research. One of them is to analyze the differences between active and passive attitude loyalty. Although some previous work in this area had shown differences between the active and passive behavior of consumers as opposed to the use of information sources (Ganesh et al., 2000; Kandampully et al., 2015), the difference between active and passive attitudinal loyalty had not been addressed. It is especially relevant to carry out work aimed at analyzing this line of research, especially given the relevance that the development of social networks can give to it.

## Author Contributions

All authors listed have made a substantial, direct and intellectual contribution to the work, and approved it for publication.

### Conflict of Interest

The authors declare that the research was conducted in the absence of any commercial or financial relationships that could be construed as a potential conflict of interest.
